# Principal function of mineralocorticoid signaling suggested by constitutive knockout of the mineralocorticoid receptor in medaka fish

**DOI:** 10.1038/srep37991

**Published:** 2016-11-29

**Authors:** Tatsuya Sakamoto, Madoka Yoshiki, Hideya Takahashi, Masayuki Yoshida, Yukiko Ogino, Toshitaka Ikeuchi, Tomoya Nakamachi, Norifumi Konno, Kouhei Matsuda, Hirotaka Sakamoto

**Affiliations:** 1 Ushimado Marine Institute, Faculty of Science, Okayama University, Setouchi, 701-4303, Japan; 2 Laboratory of Fish Physiology, Graduate School of Biosphere Science, Hiroshima University, Higashihiroshima, 739-8528, Japan; 3 Graduate School of Bioresource and Bioenvironmental Sciences, Kyushu University, Fukuoka, 819-0395, Japan; 4 Department of Bioscience, Faculty of Bioscience, Nagahama Institute of Bio-Science and Technology, Nagahama, 526-0829, Japan; 5 Laboratory of Regulatory Biology, Graduate School of Science and Engineering, University of Toyama, Toyama, 930-8555, Japan

## Abstract

As in osmoregulation, mineralocorticoid signaling is implicated in the control of brain-behavior actions. Nevertheless, the understanding of this role is limited, partly due to the mortality of mineralocorticoid receptor (MR)-knockout (KO) mice due to impaired Na^+^ reabsorption. In teleost fish, a distinct mineralocorticoid system has only been identified recently. Here, we generated a constitutive MR-KO medaka as the first adult-viable MR-KO animal, since MR expression is modest in osmoregulatory organs but high in the brain of adult medaka as for most teleosts. Hyper- and hypo-osmoregulation were normal in MR-KO medaka. When we studied the behavioral phenotypes based on the central MR localization, however, MR-KO medaka failed to track moving dots despite having an increase in acceleration of swimming. These findings reinforce previous results showing a minor role for mineralocorticoid signaling in fish osmoregulation, and provide the first convincing evidence that MR is required for normal locomotor activity in response to visual motion stimuli, but not for the recognition of these stimuli *per se*. We suggest that MR potentially integrates brain-behavioral and visual responses, which might be a conserved function of mineralocorticoid signaling through vertebrates. Importantly, this fish model allows for the possible identification of novel aspects of mineralocorticoid signaling.

Corticosteroids have two major functions in vertebrates: a glucocorticoid function that affects metabolism and growth, and a mineralocorticoid function that regulates transport of ions and water. In many vertebrates, these functions are associated with two hormones, cortisol (or corticosterone in some species) and aldosterone, which activate the glucocorticoid receptor (GR) and mineralocorticoid receptor (MR), respectively.

In teleost fish, it has long been held that a single hormone, cortisol, has both glucocorticoid and mineralocorticoid actions^1^, since fish lack aldosterone^2^,^3^. The counterparts of tetrapod MRs and their specific endogenous ligand, 11-deoxycorticosterone (DOC), have only recently been identified^4^,^5^,^6^,^7^. The effects of MR/GR agonists/antagonists and the dynamics of DOC/cortisol-MR/GR expressions indicate a minor role of mineralocorticoid signaling (DOC/cortisol-MR) in osmoregulation compared with that of glucocorticoid signaling (cortisol-GR)^8^. Thus, the function of the mineralocorticoid system in teleost fish has yet to be established.

In higher vertebrates, mineralocorticoid signaling is implicated in the control of physiological processes including brain-behavior actions, in addition to the osmoregulatory role^9^. However, understanding of this signaling is limited, partly due to the mortality of constitutive MR-knockout (KO) mice, resulting from impaired Na^+^ reabsorption at the distal tubules^10^,^11^, and the fact that a non-mouse MR-KO model has not been produced to date.

The mineralocorticoid system in fish may have biological actions in the brain and in behavior rather than in osmoregulation^8^,^12^,^13^,^14^, particularly in visuomotor performance, based on MR localization found in medaka (*Oryzias latipes*) in the present study. This fish is a useful model organism for studying these functions due to its tolerance to a wide range of salinities and several quantifiable behaviors. To better understand the role of MR, we generated the first MR-KO model, to our knowledge, in medaka using transcription activator-like effector nuclease (TALEN) KO technology. Compared with wild-type (WT) fish, MR-KO medaka show normal adaptation to fresh water and seawater, but exhibit defects in locomotor activity guided by visual motion stimuli but not in recognition of these stimuli. Our results indicate that MR is required for behaviors affected by visual stimuli, but is not essential for osmoregulation in teleost fish. These results may reveal a phylogenetically conserved link between mineralocorticoid signaling and behavior.

## Results

### Predominant MR Expression in Brain and Eyes

**Figure 1:**
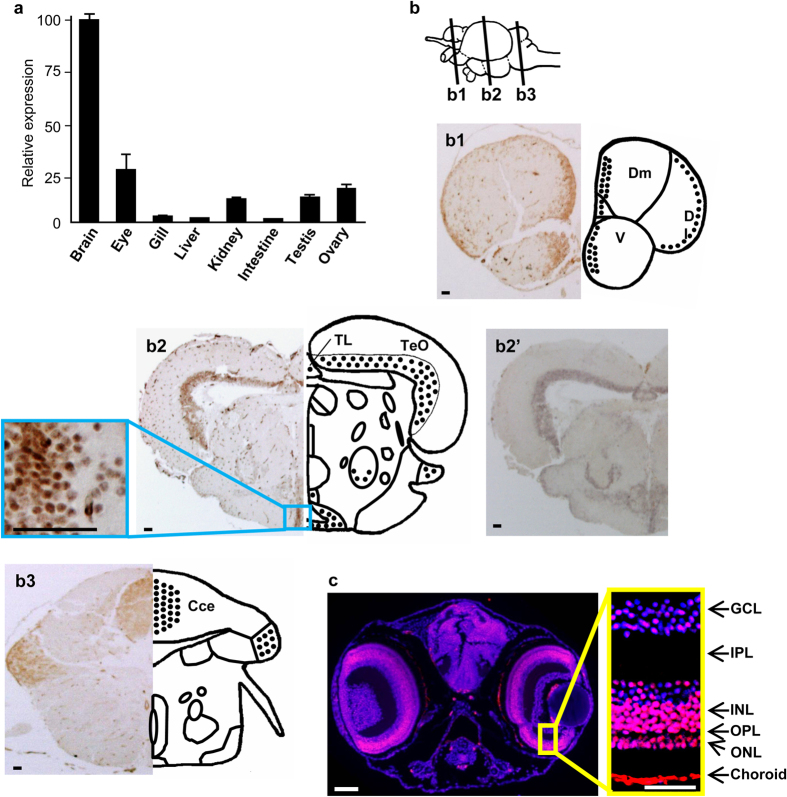
Predominant MR Expression in Brain and Eyes. (**a**) Expression of MR in tissues of adult (200 days post-fertilization) medaka determined by qPCR. Data are shown as mean ± SEM (n = 5–8 in each group). Each fish was analyzed in triplicate. (**b**) Photomicrographs and schematics of frontal sections of the medaka telencephalon (**b1**), diencephalon (**b2**) and hindbrain (**b3**), showing relatively dense (stipples) regions of MRir neuronal cell bodies. (**b2’**) *In situ* hybridization of the consecutive section using *mr* mRNA probe, showing co-localization of the signal in MRir neurons. Abbreviations: CCe, corpus cerebelli; D, dorsal telencephalic area; Dl, lateral zone of D; Dm, medial zone of D; TeO, tectum opticum; TL, torus longitudinalis; V, ventral telencephalic area. Scale bar: 30 μm. (**c**) Frontal section of medaka larvae showing MRir regions (red) in the brain and retina with nuclear staining (blue, DAPI). The larvae at 7 dpf was analyzed, since MR transcripts continuously increased 4-fold between 3 and 7 dpf, and remained at this level at 9 dpf (immediately after hatch) during early development (Supplementary Fig. 1). Strong MRir signals were observed in the nuclear layer and ganglion cell layer of the retina, as well as the choroid. ONL, outer nuclear layer (photoreceptor layer); OPL, outer plexiform layer; INL, inner nuclear layer; IPL, inner plexiform layer; GCL, retinal ganglion cell layer. Scale bar: 50 μm.

To examine the function of the mineralocorticoid system in medaka, we first assessed the *mr* expression profile. In adult medaka, MR mRNA was detected at different levels in all eight tissues examined (Fig. 1a), with the highest levels in the brain and eyes, and the lowest in the gill, liver, and intestine. The detailed expression profiles are described in Fig. 1b and c, including the first thorough description of MR expression in the CNS of an ectothermic vertebrate. Regions of the medaka adult brain that exhibited higher concentrations of MR-immunoreactive (MRir) neuronal cell bodies corresponded to homologous regions of other vertebrate brains that contain MR^12^. Regions of intense MRir neuronal populations are emphasized; for other regions with fewer MRir neurons, see Fig. 1b. Dense populations of MRir neurons were observed in the forebrain, midbrain, and hindbrain. Telencephalic regions exhibiting dense populations of MRir neurons included the ventral parts of the lateral zone of the dorsal telencephalon [Dl; putative teleostean homologue of the mammalian hippocampus^15^], and the commissural and subcommissural nuclei of the telencephalon [V; putative teleostean homologue to the mammalian amygdala^15^]. In the diencephalon, the hypothalamic preoptic area, inferior lobe of the hypothalamus, and the glomerulosus complex of the thalamus exhibited MRir neuronal cell bodies, as did the mesencephalic tegmentum and granular layer of the optic tectum. The cerebellum contained regions with prominent MRir neurons. These regions are shown in Fig. 1b. In the larval medaka head, brain had relatively dispersed MRir. Intense MRir was observed in most cells of the retina, predominantly in the nuclear and ganglion cell layers and choroid (Fig. 1c). Thus, the presence of MR-positive cells in regions that are important for visuomotor performance, including the dorsal part of the telencephalon, optic tectum (torus longitudinalis), cerebellum, and retina, is of particular interest^16^,^17^. There were no differences in expression between males and females.

### Generation and Identification of *mr* Mutant Alleles

**Figure 2:**
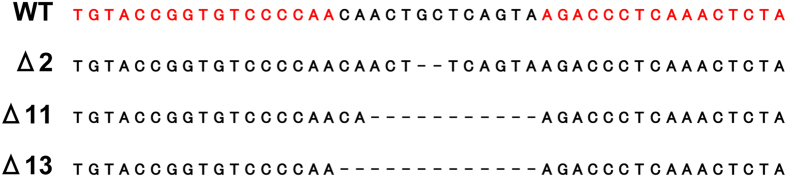
TALEN design and results of indel mutations in medaka *mr*. Alignment of WT *mr* sequence with mutated PCR amplicons. A pair of TALEN was designed near the start ATG codon. The target sequences of the TAL effector DNA-binding domains are highlighted in red. Δ2, Δ11 and Δ13 alleles with 2–11- and 13-nucleotide deletions, respectively.

Two TALEN arms were designed targeting exon 1 of the medaka *mr* gene, centered near the ATG start codon (Fig. 2). TALEN RNAs (25 pg left + 25 pg right) were injected into fertilized eggs at the 1 cell stage to produce *mr* mutant alleles. Bacterial clones of PCR-amplified genomic DNA isolated from 2–3 dpf TALEN-injected embryos revealed indel mutations in the target region, ranging from a 1 to 13 nucleotide deletion, but no insertion. Sibling embryos were raised to 2 months and screened individually by heteroduplex mobility assay (HMA) using fin biopsy genomic DNA, which identified 13 out of 68 fish carrying somatic indel mutations. These heterozygous carriers (XX females) were out-crossed with WT fish of an inbred strain, Hd-rR (XY), and offspring (F1) were raised at a reduced culturing density (6 fish per 1.8 L) to accelerate growth, and allow breeding as early as 2 months. A HMA of 1-month-old F1 fish revealed an average of 10% germline transmission of mutant alleles, with the same mutant allele present in all individuals derived from a single G0 parent. Deletion mutations of 2, 11 and 13 nucleotides were identified (Δ2, Δ11 and Δ13; Fig. 2). The Δ2 mutant alleles were identified first, and for this reason we focused on this line for subsequent analyses. DNA sequence genotyping showed that the *mr*Δ2 mutant allele generates a frameshift at amino acid #42, followed by a missense polypeptide of 7 amino acids that shows no significant similarity to any putative protein in the NCBI database. Wild-type medaka MR has 994 amino acids (108 kDa)^18^.

In F1 generation, all *mr*Δ2 heterozygous carriers turned out to be male. Δ2 males were crossed with Hd-rR females to obtain Δ2 females (F2), which were identified by RFLP genotyping. F2 Δ2 females were crossed with F1 Δ2 males to obtain a pool of offspring yielding the expected Mendelian proportion of homozygous and heterozygous *mr*Δ2 mutants, as determined by RFLP and DNA sequence genotyping. Juvenile *mr*Δ2 individuals were raised and in-crossed to obtain next-generation homozygous mutants, which were used for further experiments. We refer to these populations as MR-KO. Western blot analysis of total protein using the medaka MR antibody showed that MR was expressed in WT fish, but not in MR-KO fish, indicating that MR was successfully knocked out.

### Normal Physical Characterization of MR-KO Medaka

**Figure 3:**
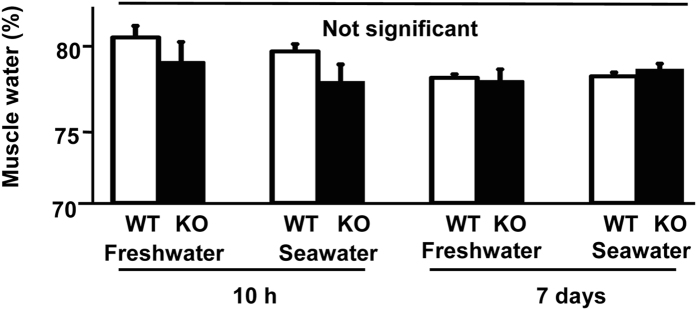
Muscle water content in MR-KO adult medaka in fresh water and after seawater transfer. MR-KO was confirmed by fin biopsy genotyping. Data are shown as mean + SEM (n = 7). There was no significant difference for each time period (P > 0.05).

MR-KO embryos, juveniles and sexually mature adults of medaka exhibited no signs of physical abnormalities, including in retinal cell layers assessed by gross anatomical measurements and hematoxylin and eosin staining histology. This is in contrast with MR-KO mice, in which mortality results from impaired osmoregulation. The muscle water content of MR-KO medaka was analyzed in fresh water and after seawater transfer (10 h and 1 week). Our previous study of seawater transfer showed a water loss from muscles of medaka by 2 h, followed by tissue rehydration to the original level by 10 h and normalized within 1 week^19^. Muscle water content in MR-KO adult medaka was normal compared with WT counterparts in fresh water and after seawater transfer (Fig. 3). Therefore, we conclude that, unlike in mammals, osmoregulation is normal in MR-KO medaka.

### MR-KO Medaka have Abnormal Responses to Visual Motion Stimuli

**Figure 4:**
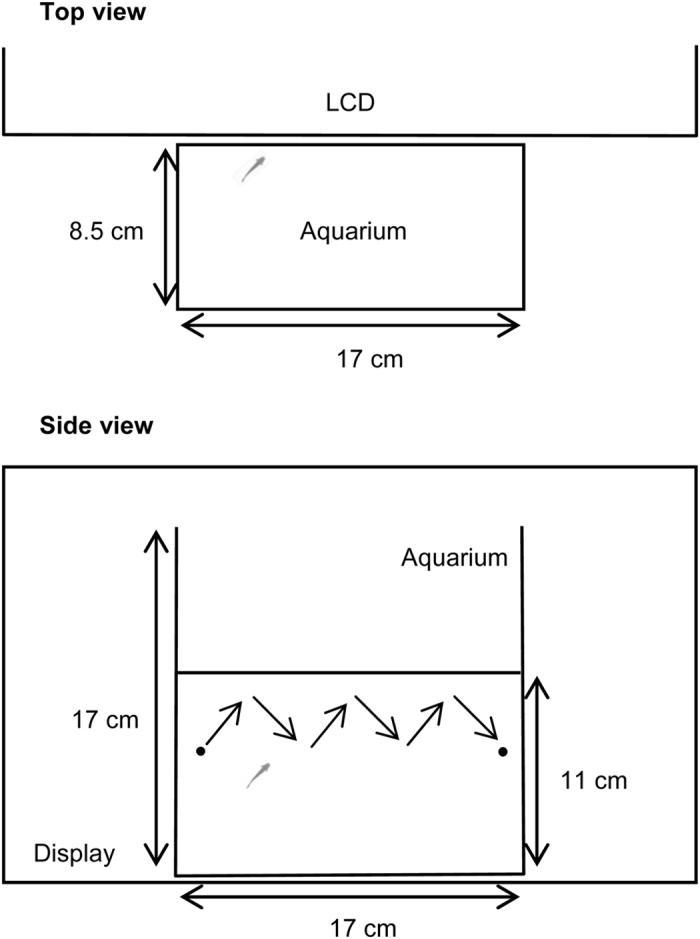
Schematic of the experimental set-up for the behavioral test. Stimuli were presented within an area of 615 × 72 pixels (170 × 20 mm^2^) located in the center of a 24-inch liquid-crystal display with a refresh rate of 60 Hz and resolution of 1,920 × 1080 pixels, and were controlled by computer software. The tip of the head of the fish and the dot was tracked using computer software. The distance to the dot and acceleration of the fish were analyzed.

**Figure 5:**
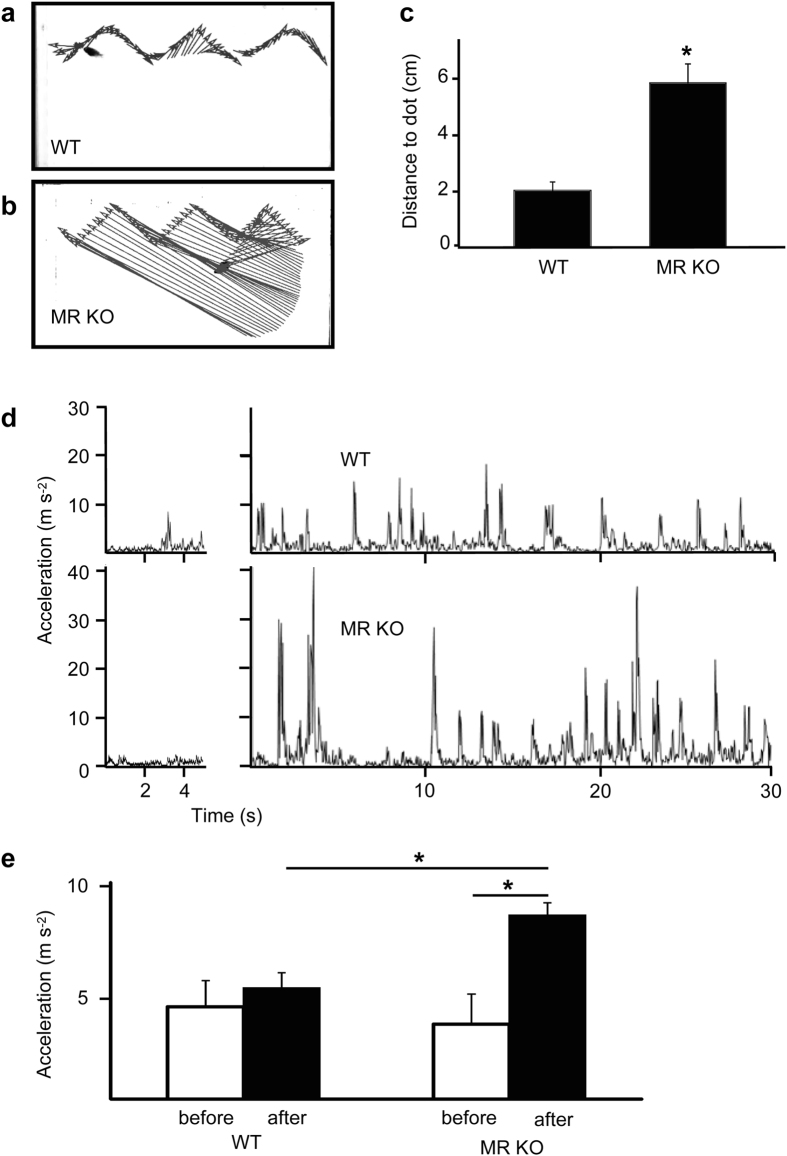
Abnormal responses to visual motion stimuli in MR-KO medaka. Naive fish were used in the behavioral test (*n* = 6). (**a**) Distance to the stimulus dot from the head and trajectory of a representative WT fish during stimulus presentation. (**b**) Distance to the dot from the head and trajectory of a representative MR-KO medaka during stimulus presentation. (**c**) Averaged distance from the head to the stimulus dot. (**d**) Acceleration during observation. The first 30 s at baseline (left) and the first 15 s and last 2.25 min of the stimulus presentation period (right) are not included. The WT fish (upper) swam at approximately constant speed, whereas the MR-KO fish (lower) swam in spurts during stimulus presentation. (**e**) Acceleration averaged during periods at baseline (before) and in stimulus presentation (after). The MR-KO fish swam significantly more than the WT fish during stimulus presentation, but the acceleration before stimulus presentation is similar in the two groups. Error bars show ± SEM.

Based on MR expression in medaka brain and eyes, responses of WT and MR-KO medaka to movements of black dots were recorded to test if MR-KO fish showed abnormal behavior (Fig. 4, Supplementary Video 1)^20^. The coordinates of the head of the fish were tracked in each video frame, and the distance from the head to the dot and acceleration were measured (Fig. 5). Before presentation of the moving dot, both WT and MR-KO fish swam moderately, with no detectable genotype difference (Fig. 5d; compare left upper and lower panels) after resuming normal swimming. When moving dots were presented, there were significant differences in the behavioral patterns of MR-KO and WT fish. The WT fish tracked the dot and the MR-KO fish showed inferior tracking ability. Thus, the distance to the dot for MR-KO fish was significantly greater than that for WT (Fig. 5c). Regarding acceleration during stimulus presentation, tracking by WT fish was smooth (Fig. 5d; right upper panel), whereas MR-KO fish showed jerky swimming episodes with bouts across the tank (Fig. 5d; right lower panel). During stimulation, a significant increase in acceleration from baseline was found only in MR-KO fish, and this increased acceleration was greater than that for WT fish (Fig. 5e). These results suggest that MR-KO medaka can recognize a stimulus, but that responses to the stimulus were abnormal.

## Discussion

It is well-established that mineralocorticoid signaling is required for sodium homeostasis in mammals^21^, but the function in fish is unclear^4^,^8^. Studies using treatment with ligands, analysis of endogenous ligand-MR dynamics and knockdown of MR have led to somewhat contradictory results^8^,^18^,^22^,^23^,^24^,^25^. Loss-of-function and KO studies are currently lacking in non-mammalian models. Using TALEN technology, we generated the first constitutive MR-KO model in a vertebrate, and here we provide the first evidence that MR is required for normal behavior during exposure to visual motion stimuli, rather than for osmoregulation.

It is generally accepted that MRs in the mammalian brain are involved in physiological processes such as limbic system and hypothalamus pituitary adrenal (HPA) axis activities^9^,^26^. Behavioral results in mammals, however, are inconsistent, and hamper advancement in the field of mineralocorticoid signaling and actions. To date, these studies have relied heavily on a few mouse lines with conditionally altered MR expression or function^27^,^28^ (constitutive KO of MR is neonatal lethal in mouse), and tissue-restricted manipulations have incomplete or complex effects. Thus, it is critical to establish KO models with null alleles for MR genes and to study the effects on these processes. In homozygous MR-KO medaka, we observed defects in behavior during exposure to visual stimuli, and therefore we conclude that MR plays an essential role in these behaviors, and not in osmoregulation.

In osmoregulation in fish, cortisol acting via the GR may play a more important role than mineralocorticoid signaling^8^. Cortisol induces Na^+^ -K^+^-ATPase in Atlantic salmon and improves salinity tolerance via the GR, whereas DOC and aldosterone (which are ligands for teleost MR but not for GR) had no effect^29^. Also, Trayer *et al.*^18^ and Cruz *et al.*^22^ found that antisense knockdown of GR, but not of MR, downregulated differentiation of ionocytes in medaka and zebrafish. In our studies on the osmoregulatory esophagus of teleosts, cortisol appears to have a direct GR-dependent effect on differentiation of the esophageal epithelium during acclimation to different salinities, while DOC does not show any significant effects^30^,^31^. Furthermore, expression of GR mRNA is generally higher than that of MR mRNA in peripheral tissues, including osmoregulatory organs, but levels of MR and GR are similar in cichlid fish (*Astatotilapia burtoni*) and midshipman brain^7^,^32^. We (Fig. 1a) and Sturm *et al.*^6^ have examined many tissues in rainbow trout and medaka, and found that expression of MR mRNA is highest in brain and eyes. MR transcripts increase at hatch toward onset of visuomotor performance^33^ in the head (see Supplementary Fig. 1). The results in this study showing that MR-KO fish adapt to both fresh water and seawater provide clear evidence that MR is not required for osmoregulation in medaka.

Critical roles of MR in neuroendocrine functions, including stress, anxiety and cognition, have been shown in mammals using brain MR antagonists and in mouse with conditionally altered MR expression^28^,^34^. Blockade or KO of brain MR impairs memory and the HPA axis^27^,^35^,^36^,^37^,^38^,^39^,^40^,^41^, whereas transgenic MR overexpression in forebrain dampens anxiety-related behavior in mice^26^. In the current study, defects in behavior of MR-KO medaka during exposure to visual stimuli have been observed, possibly due to damage in vision and brain-dependent behavior, as described for a zebrafish *gr* mutant^42^,^43^. Such damage may modulate the neuroendocrine functions mentioned above, and thus our results may indicate a primarily conserved function of MR among vertebrates. Corticosteroids could integrate the HPA axis and limbic system with visual physiology of the sensory periphery, as part of a whole-organism coping mechanism that facilitates adjustment to rapid environmental changes, including visual stimuli. Our MR-KO medaka model thus provides a unique opportunity for identifying conserved networks regulated directly by MR in vertebrates.

The terms “glucocorticoid” and “mineralocorticoid” have their origin in mammalian studies. The results of this study showing essential MR function in brain-dependent behaviors of a teleost, and those of previous studies, suggest that these terms may be less appropriate, at least for teleost fish. The kidney action of mammalian mineralocorticoid signaling might have been acquired during evolution of the loop of Henle, a well-known mineralocorticoid target in mammals^44^, whereas brain-dependent behavioral function observed also in higher vertebrates may reflect the principal MR function through vertebrates. The evolution of the role of corticosteroids is intriguing. Since the MR is thought to be ancestral to the GR^45^,^46^, this principal function of MR may also reflect the original function of corticosteroid signaling.

## Methods

### Fish

The Hd-rR strain was used in the study. Handling and care was carried out using standard procedures^47^ in accordance with the Animal Research Guidelines at Okayama University. All procedures were approved by the Committee for Animal Research, Okayama University. All fish were raised and reared in aged tap water at 27 ± 2 °C with a 14:10 h light:dark cycle.

### Gene Expression of *mr* by qRT-PCR

Total RNA from brain, eyes, gill, liver, kidney, intestine, testis and ovary of adult Hd-rR was extracted using a RNeasy Plus mini kit (Qiagen). RNA quantity was determined by a Qubit Fluorometer (Invitrogen). Total RNA was reverse transcribed using the Omniscript RT Kit (Qiagen). qRT-PCR was performed using an ABI Prism 7000 (Applied Biosystems) and SYBR Premix Ex Taq II (Takara)^48^,^49^. Standard operating conditions were 95 °C, 30 s, followed by 40 cycles at 95 °C for 5 s and 60 °C for 31 s. In each experiment, housekeeping genes RPL-7 (*rpl7*) were quantified concurrently. Gene-specific primers were designed [*mr*: 5′-CCA GAG GTG AAG GGT ATC CA-3′ and 5′-GAA GCC TCG TCT CCA CAA AC-3′; for *rpl7*^48^]. The dissociation curves of the primer pairs showed a single peak. Relative standard curves were constructed for *mr* and *rpl7* using cDNA stock from medaka RNA. The *rpl7* mRNA level was relatively constant, as also shown previously^48^,^49^,^50^. Results for *mr* were normalized using *rpl7* mRNA levels.

### Localization of MR by Immunohistochemistry and *in situ* hybridization

Medaka adult brains and larvae at 7 dpf were fixed in 4% paraformaldehyde prepared in phosphate-buffered saline (PBS) at 4 °C overnight, since MR transcripts continuously increased 4-fold between 3 and 7 dpf, and remained at this level at 9 dpf (immediately after hatch) during early development (see Supplementary Fig. 1). Fixed tissue samples were dehydrated through graded alcohol, embedded in paraffin wax, and serially sectioned at 8 μm. Frontal sections were placed on MAS-GP coated slides. Brains and larvae were subjected to immunostaining by the avidin-biotin peroxidase complex (ABC) method^51^ using Vectastain ABC Kit (Vector Laboratories, Burlingame, CA) and to fluorescent immunostaining, respectively, as described before^31^,^52^,^53^. The slides were subjected to heat-induced antigen retrieval by heating at 90 °C in target retrieval solution for 20 min (Dako). Subsequently, sections were washed in 0.3% Triton-X in PBS (PBST) and placed in a solution containing 1% normal goat serum and 1% BSA in PBST at room temperature for 30 min to block non-specific binding. Sections were incubated at 4 °C for 2 days with MR antiserum (1:2000). This antibody was raised against a peptide specific for the A/B domain of medaka MR (DSKDNNSNKQEQPRLQL; Medical & Biological Laboratories, Japan), which shares low sequence identity (<20%) with other steroid receptors^2^,^18^. Pre-absorption of the antibody with the antigen peptide completely prevented staining in immunohistochemistry. Furthermore, cellular localization of the immunoreactive MR correlated with the respective cellular expression of the *mr* gene identified by *in situ* hybridization carried out using the DIG-labeled antisense riboprobes (Fig. 1b2) as described^54^. Images were captured with an Olympus FSX100 fluorescent microscope. The MR antibody specificity was also confirmed by performing western blots on protein extracts from brains^55^.

### Design and Generation of TALENs

TALEN reagents used in this study were designed and constructed by Transposagen. Briefly, candidate TALEN target sequences in the medaka *mr* gene retrieved from the Ensemble medaka genomic database (http://www.ensembl.org/Oryzias_latipes/index.html) were identified, and from these we selected one near the start methionine codon to achieve maximal disruption of the *mr* coding sequence (see Fig. 2 for positions and target sequences). Initial validation tests for cleavage activity were performed on episomal target sequences *in vitro* by Transposagen.

For expression in medaka embryos, TALEN RNAs were synthesized *in vitro* using a mMessage mMachine T7 Ultra kit (Life Technologies) and injected into Hd-rR embryos. TALEN mRNAs (25 pg of each arm: the dosage giving the highest indel mutation efficiency and <50% morphological toxicity at 24 hpf) were injected at the 1-cell stage. To detect indel mutations in the TALEN target region, HMA was performed^56^,^57^,^58^. Crude extracts of genomic DNA were prepared from medaka embryos at 2–3 dpf using KAPA Express Extract (Nippon Genetics). 1 μL of this crude extract was used as template for subsequent PCR. A 264-bp amplicon flanking the TALEN target region was amplified with the primer pair (forward 5′-TGT CCA GCC CTC ACA GTA TG-3′; reverse 5′-GGC TGC TGC TAT CGT TCT G-3′^58^ using Emerald Amp PCR Master Mix (Takara) in a Thermal Cycler (BioRad). The reaction profile consisted of polymerase activation at 95 °C for 3 min, followed by 35 cycles of denaturation at 98 °C for 10 s, annealing at 60 °C for 30 s and extension at 72 °C for 1 min, with final extension at 72 °C for 5 min. The resulting amplicons were analyzed using discontinuous polyacrylamide gels with a 4% stacking gel and a 9% separating gel for the HMA. When a heteroduplex polymorphism was observed, the PCR amplicon was cloned into the pMD-20 vector using a Mighty TA cloning kit (Takara) and sequenced (Macrogen).

### Genotype Analysis by RFLP

Genomic DNA from tail fin biopsies or whole embryos was prepared and the region containing the target site of the TALEN was amplified, as described above. The resulting PCR products that were cloned and sequenced for verification were digested at 37 °C for 30 min in 20 μL of DdeI restriction digestion solution that consisted of reaction buffer and 2 units of DdeI (New England Biolabs). The 264 bp PCR product was cut to yield 126 bp and 138 bp fragments for only WT alleles. These digestion products were analyzed using agarose gels.

### Analysis of Osmoregulation

Muscle water content was used as an indicator of osmoregulatory capacity because it is difficult to collect blood from small medaka. In many teleost species, including medaka, changes in muscle water content are inversely related to changes in plasma osmolality, sodium and chloride^19^,^59^,^60^,^61^,^62^,^63^. Adult medaka in fresh water (0 ppt) were transferred to fresh water or seawater in the morning (0–1 h after light onset; 0700–800 h). After 10 h and 1 week transfer to seawater, the caudal portion of the paraxial muscle was dissected out. Samples of muscle were immediately weighed. Dry weights were obtained after drying at 100 °C until constant weight was attained (24 h), and the water content was expressed as a percentage of the wet weight.

### Behavioral Tests and Image Analysis

Plastic aquaria attached to the LCD (U2410, Dell) (Fig. 4) were used for the behavioral test. To limit visual stimulation, the lateral sides were covered with white polystyrene sheets. The test tanks were filled with housing water (water depth 11.0 cm) maintained at a 27 ± 1 °C by air conditioning. To the test aquarium, a fish which was naive to the aquarium and randomly selected from the WT or MR-KO group was individually transferred. Eighteen hours later, the LCD was attached to the tank and the animal was allowed to habituate for 15 min. The behavioral test consisted of a baseline period (1 min) and a stimulus presentation period (3 min). Illumination was adjusted with white florescent lamps on the ceiling. After the habituation period, the behavior of the fish was recorded for 3.5 min. All behavioral testing was restricted to the early afternoon (6–9 h after light onset; 1300–1600 h). The first 1 min of recording was used to determine the baseline activity, in which no stimuli were presented. Visual motion stimuli were presented on the LCD during the last 3 min (stimulus presentation period). The stimuli were animations of a zigzagging black dot (1 mm in diameter) on a white background, controlled by a program written in LabVIEW 8 (National Instruments, Austin, TX, USA) running on a Windows PC. Stimuli were presented as 3-min animations constituting six repetitions of the same 30-sec animation (Fig. 4, Supplementary Video 1). This design was used because preliminary studies showed that recognition/tracking by WT medaka was maximized at this speed and decreased at faster (2 and 1.5 times) and slower (0.5 and 0.25 times) speeds.

Movies were recorded at a frame rate of 30 fps with a digital camera (HandyCam, Sony Electronics) and saved on the hard disk. Offline analysis of the original sequence (all frames) was carried out with video analysis software (Tracker ver. 4.86, http://physlets.org/tracker/). Coordinates of the head of the fish were tracked in each video frame (Fig. 5a,b) and the distance from the head to the dot was measured. The trajectory of the fish was graphed over time and evaluated with respect to acceleration (Fig. 5d). Due to the dimensional limits, the behavior was assessed using two-dimensional coordinates from the side view.

### Statistics

Statistical analysis was carried out with Statview 5.0 (Abacus Concepts) using one-, two- or three-way analysis of variance, followed by appropriate post-hoc tests. Data were checked for normality and equal variances.

## Additional Information

**How to cite this article**: Sakamoto, T. *et al.* Principal function of mineralocorticoid signaling suggested by constitutive knockout of the mineralocorticoid receptor in medaka fish. *Sci. Rep.* **6**, 37991; doi: 10.1038/srep37991 (2016).

**Publisher's note:** Springer Nature remains neutral with regard to jurisdictional claims in published maps and institutional affiliations.

## Supplementary Material

Supplementary Information

Supplementary Video 1
