# Supplementary material for: Principal function of mineralocorticoid signaling suggested by constitutive knockout of the mineralocorticoid receptor in medaka fish
Source: Sci Rep. 2016 Nov 29;6:37991. doi: 10.1038/srep37991 (PMC5126551; doi:10.1038/srep37991)
Supplement: Supplementary Information [file srep37991-s1.doc]

**Supplementary Information:**

**Principal function of mineralocorticoid signaling suggested by constitutive knockout of the mineralocorticoid receptor in medaka fish**

Correspondence: ryu@uml.okayama-u.ac.jp

Tatsuya Sakamoto ^1^ *, Madoka Yoshiki ^1^, Hideya Takahashi ^1^, Masayuki Yoshida ^2^, Yukiko Ogino ^3^, Toshitaka Ikeuchi ^4^, Tomoya Nakamachi ^5^, Norifumi Konno ^5^, Kouhei Matsuda ^5^, Hirotaka Sakamoto ^1^

1 Ushimado Marine Institute, Faculty of Science, Okayama University, Setouchi 701-4303, Japan

2 Laboratory of Fish Physiology, Graduate School of Biosphere Science, Hiroshima University, Higashihiroshima 739-8528, Japan

3 Graduate School of Bioresource and Bioenvironmental Sciences, Kyushu University, Fukuoka 819-0395, Japan

4 Department of Bioscience, Faculty of Bioscience, Nagahama Institute of Bio-Science and Technology, Nagahama 526-0829, Japan.

5 Laboratory of Regulatory Biology, Graduate School of Science and Engineering, University of Toyama, Toyama 930-8555, Japan

**Supplementary Information**

**Supplementary Figure 1.** Expression of MR in medaka embryos and larvae from 3 to 9 days post-fertilization (dpf), as determined by qPCR.

**Supplementary Video-1** **Title and Legend.**

**Supplementary Figure 1. Expression of MR in medaka embryos and larvae from 3 to 9 days post-fertilization (dpf), as determined by qPCR.** Food was first offered at 9 dpf. Values are means ± SEM (n = 5-10). Symbols with different letters (A, B, C) are significantly different, as determined by ANOVA (P < 0.01) followed by Tukey post hoc comparison of differences among groups. When total RNA was extracted from the head and body trunk with the gill at 7 dpf, MR was primarily expressed in the head.

**Supplementary Video 1. Medaka responding to movements of black dots.** Dot-tracking by the WT fish was nearly perfect and markedly smooth, whereas MR-KO fish showed inferior tracking ability and jerky swimming episodes with bouts across the tank during presentation of the dot. Genotype differences were not detectable before dot presentation.
